# Cortical Classification with Rhythm Entropy for Error Processing in Cocktail Party Environment Based on Scalp EEG Recording

**DOI:** 10.1038/s41598-018-24535-4

**Published:** 2018-04-17

**Authors:** Yin Tian, Wei Xu, Li Yang

**Affiliations:** Bio-information College, ChongQing University of Posts and Telecommunications, ChongQing, 400065 China

## Abstract

Using single-trial cortical signals calculated by weighted minimum norm solution estimation (WMNE), the present study explored a feature extraction method based on rhythm entropy to classify the scalp electroencephalography (EEG) signals of error response from that of correct response during performing auditory-track tasks in cocktail party environment. The classification rate achieved 89.7% with single-trial (≈700 ms) when using support vector machine(SVM) with the leave-one-out-cross-validation (LOOCV). And high discriminative regions mainly distributed at the medial frontal cortex (MFC), the left supplementary motor area (lSMA) and the right supplementary motor area (rSMA). The mean entropy value for error trials was significantly lower than that for correct trials in the discriminative cortices. By time-varying network analysis, different information flows changed among these discriminative regions with time, i.e. error processing showed a left-bias information flow, and correct processing presented a right-bias information flow. These findings revealed that the rhythm information based on single cortical signals could be well used to describe characteristics of error-related EEG signals and further provided a novel application about auditory attention for brain computer interfaces (BCIs).

## Introduction

In everyday life, the flood of sensory information were regulated by attention system into a manageable stream, and attention orienting played a primary role in complex visual environment by finding relevant information and filtering out irrelevant information to bias the target selection and processing^1^. Typically, two mechanisms were thought to be included in the process: endogenous orienting (goal-driven, top-down), directed the attention to the information related locations in space, and exogenous orienting (stimulus-driven, bottom-up), reflexively triggered by prominent and behaviorally relevant stimuli^2^. Classic research on attention orienting was involved by the analysis of the cocktail party phenomenon coined by Cherry in 1953^3^.

The cocktail party effect was the phenomenon that people can focus their auditory attention on a stimulus while filtering out other stimuli, similar with a partygoer being able to concentrate on a single conversation in a noisy room, namely, the process reflected the influence of top-down attention. It might also describe a similar phenomenon that occurs when one can immediately detect words of importance originating from unattended stimuli, for instance hearing one’s name in another conversation, which referred to the bottom-up controlled attention^4–6^.

A lot of researches was conducted to investigate dynamic changes in cortical activity during tracking the dynamic speech stimulus^4,5^, and the findings suggested that attentional orienting modulated the neural responses to one of speakers’ voices. If a listener successfully tracked one speaker in a multi-speakers’ environment, the neural responses showed highly correlated with the attended speaker^4,7–9^. And the neural generator of this effect was localized in the left hemisphere^10^. However, when people were absent-minded, they often failed to keep track of the goals that needed to be noticed; that is, the wrong execution was mostly due to the lack of attention to the target stimulus.

Over the last decade, a lot of researches focused on the theories of attention orienting^11^. In contrast, little is known about the connection of attention with BCIs. Several recent studies have investigated the impact of attention on BCIs. A typical example was to utilize attentional modulation of steady-state visual evoked potential (SSVEP) to implement an online BCI system, and the results showed the SSVEP amplitude could be enhanced by attention, thus improving the speed and accuracy of the BCI system^12^. Another work researched SSVEP under strong attention and poor attention of flash conditions, the results indicated that the SSVEP was modulated by attention and the effect of modulations was related to the frequency of flash stimulation^13^. Further, the simultaneously presented tactile and visual stimuli were used to investigate the influence of attention shift on SSVEP. The significant attention switching was observed within both types of stimuli and between different stimuli. Similarly, the unattended experiments were also investigated in the BCI studies. For instance, the error-related negative (ERN) potential reflected an error-monitoring process of the brain and could be detected in scalp EEG recordings^14,15^. The ERN arose after an erroneous response and the maximum peak was localized at the medial frontal regions^14,16^. Recently, researchers found that the ERN potential was used in BCI for adjusting command outputs of BCI systems when subjects observed incorrect outputs from BCI systems, thus facilitating the development of BCI systems with improved accuracy^17^.

Although BCI studies regarding attention have been conducted, some shortcomings still existed. Firstly, methods, which were used to select feature from the EEG signals, were not related to the cognitive functions. For example, information entropy described the generating rate of new information of nonlinear dynamical systems and it has been shown as an effective measure to select EEG signals features^18^. However, information entropy ignored the association between EEG activities and subjects’ cognitive states. Consequently, a similar order state of EEG signal sequence could be found among different cognitive states, which hampered the applications in clinical areas^19^. Secondly, the BCI performance was limited by the measurement manners of EEG. Compared with the intracranial EEG, which was directly recorded from the cortex surface, the scalp EEG could be easily affected by the effect of volume conduction and reference electrodes^20–22^, causing an imprecise measurement of physiological significance. Although the intracranial EEG described more precise temporal and spatial information than that of the scalp EEG, it was invasive and only feasible for a very limited number of subjects^21,23^. Thirdly, the regional EEG parameters could not adequately reflect the cognitive process. Multiple brain regions were involved in the cognitive processing and reflected by the EEG activities^24^. Recently, network analysis methods have attracted wide-spread attention in neuroscience and it proved to be an efficient way to measure the connections between regions in the cognitive functions^25,26^.

In the present study, the cocktail party experiment paradigm, which was closer to the real environment, was used to research the error-related attention. In order to overcome the drawbacks of previous studies on the scalp EEG, the weight minimum norm (WMN) method was used to estimate cortical activities with single-trial. Then, we adopted a feature extraction method, rhythm entropy, to classify the error-related auditory processing from correct auditory processing based on single cortical signals. Rhythm entropy (RhEn) was developed by combining the information entropy with the power of EEG rhythm, which was an important feature for cognitive research based on spontaneous EEG^27^. Finally, adaptive directed transfer function (ADTF), one of the most frequently used methods for assessing the dynamic causality relationship among various brain regions^28,29^, was applied to calculate the time-varying connectivity patterns in the different conditions.

## Result

### Reaction Time (RT)

**Figure 1:**
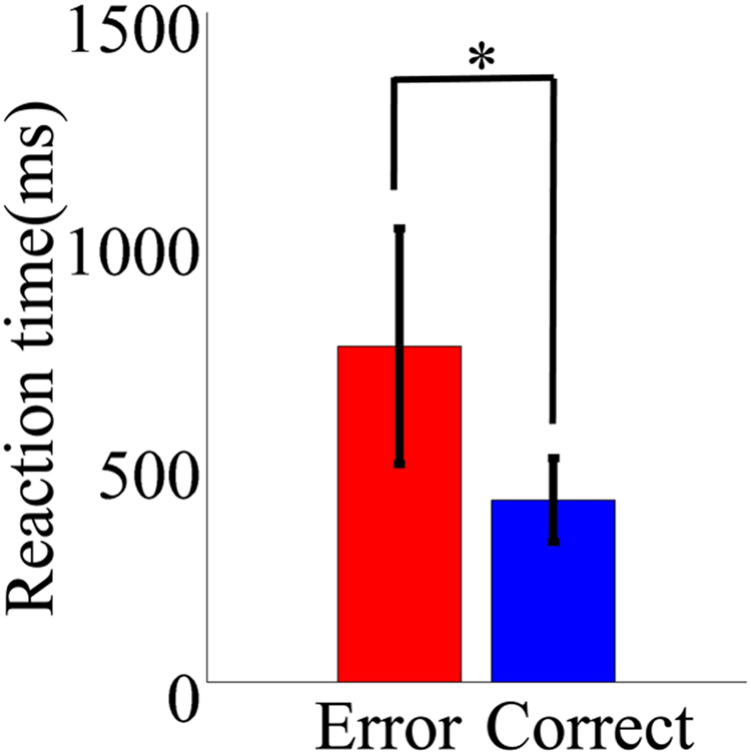
Mean (with SD) reaction time (RT) of subjects.

As shown in Fig. 1, mean RTs for correct response were shorter than those for error response (paired t-test: t = −7.2, *p* < 0.05, d = −0.66).

### Brain regions with high discriminative power

**Figure 2:**
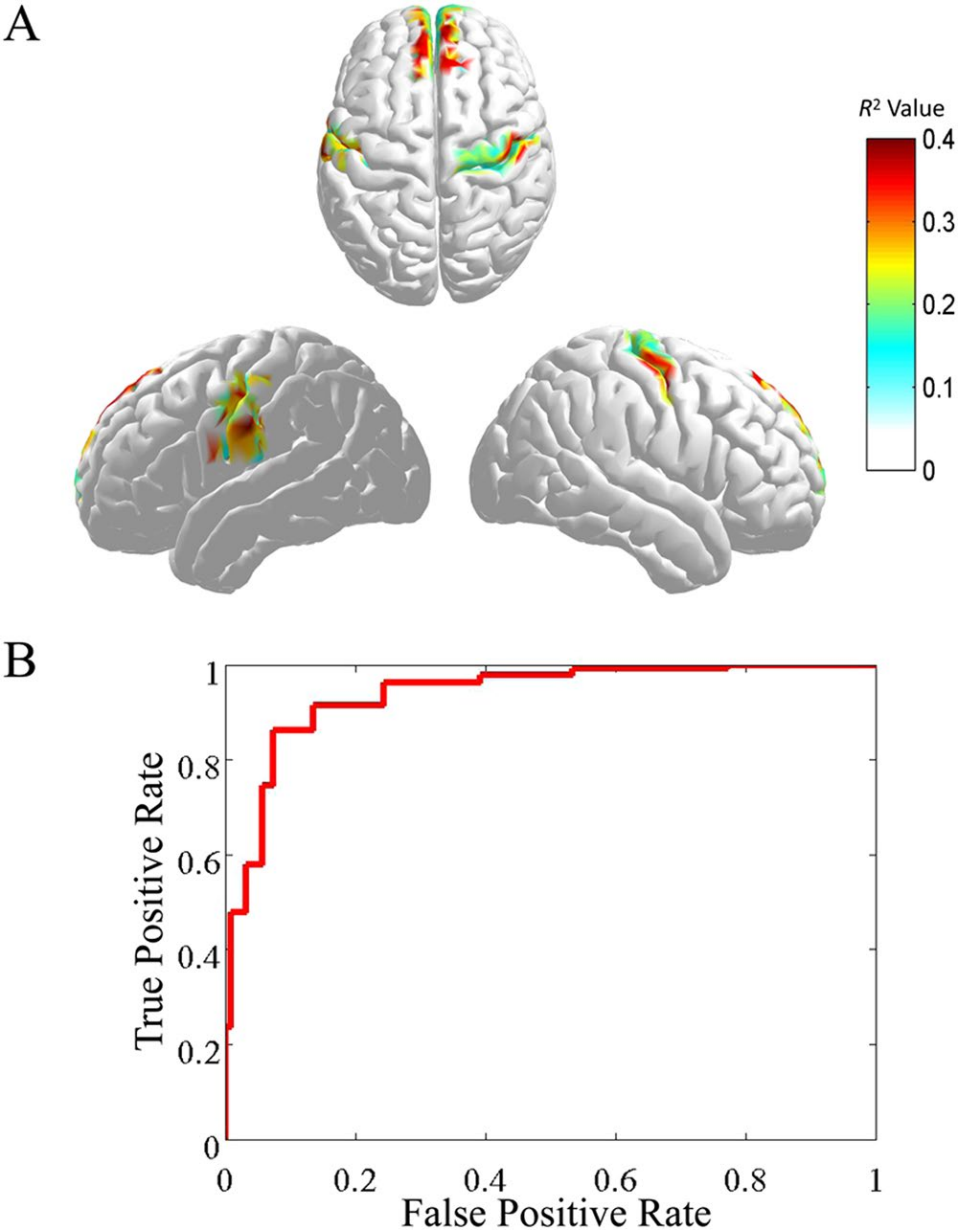
The discriminative sources and ROC curve. (**A**) The projection of mean R^2^ values of 20 subjects on cortical regions; (**B**) Mean ROC curve for 20 subjects.

For visual representation, the cortical spatial distribution was reconstructed according to the *R*^2^ value on each dipole. Three brain regions, i.e. medial frontal cortex (MFC), left supplementary motor area (lSMA) and right supplementary motor area (rSMA), exhibited greater correlation than others (Fig. 2A and Table 1), got high discriminative power for identifying error response trials from correct response trials during tracking the cued speaker.

**Table 1:** Brain regions with high discriminative power.

Brain region	L/R	BA	Number of dipoles	Mean R^2^	MNI Coordinates		
					x	y	z
Medial frontal cortex (MFC)							
Medial superior frontal gyrus	L	BA8	6	0.360	−8	40	54
	R	BA8	7	0.350	8	50	48
Middle frontal gyrus	L	N/A	7	0.363	−25	9	56
	R	BA6	6	0.346	44	0	56
Superior frontal gyrus	L	BA8	12	0.384	−14	38	56
	R	BA9	13	0.347	22	56	36
Anterior Cingulum Cortex	L	BA32	2	0.328	−4	30	22
Left supplementary motor area (lSMA)							
Postcentral Gyrus	L	BA4	5	0.350	−44	−16	54
Precentral Gyrus	L	BA4	6	0.326	−38	−22	46
Supplementary motor area	L	BA6	6	0.324	−10	24	60
Insula	L	N/A	3	0.323	−34	−12	16
Inferior parietal lobule	L	BA2	3	0.355	−58	−26	50
Right supplementary motor area (rSMA)							
Precentral Gyrus	R	N/A	1	0.341	48	−8	52
Postcentral Gyrus	R	BA3	5	0.341	56	−14	54
Supplementary motor area	R	BA6	5	0.325	4	6	56
Superior temporal gyrus	R	BA40	1	0.345	66	−20	14

### Classification accuracy

The SVM with LOOCV achieved an average accuracy of 89.7%(SD ± 3.6%). A good generalization performance of SVM classifier were observed (Table 2 and Fig. 2B), i.e. SP: 89.6% ± 4.9%, SE: 89.8% ± 5.2%, AUC: 94.0% ± 5.2%.

**Table 2:** Classification results of SVM classifier.

Subject	CA	SP	SE	AUC
1	0.893	0.857	0.929	0.952
2	0.893	0.929	0.857	0.967
3	0.833	0.800	0.867	0.804
4	0.889	0.833	0.944	0.965
5	0.933	0.933	0.933	0.931
6	0.882	0.941	0.824	0.891
7	0.921	0.895	0.947	0.922
8	0.906	0.938	0.875	0.972
9	0.969	0.941	0.987	0.981
10	0.933	0.892	0.975	0.998
11	0.900	0.933	0.867	0.929
12	0.893	0.857	0.929	0.965
13	0.886	0.909	0.864	0.934
14	0.857	0.809	0.905	0.981
15	0.923	0.896	0.950	0.884
16	0.821	0.857	0.786	0.996
17	0.933	0.980	0.887	0.998
18	0.885	0.923	0.846	0.846
19	0.923	0.932	0.914	0.953
20	0.864	0.864	0.864	0.935
Mean	0.897	0.896	0.898	0.940

Note: CA represents classification accuracy, SP represents specificity, SE represents sensitivity and AUC represents area under ROC curve.

### Relationship between RT and rhythm entropy (RhEn)

**Figure 3:**
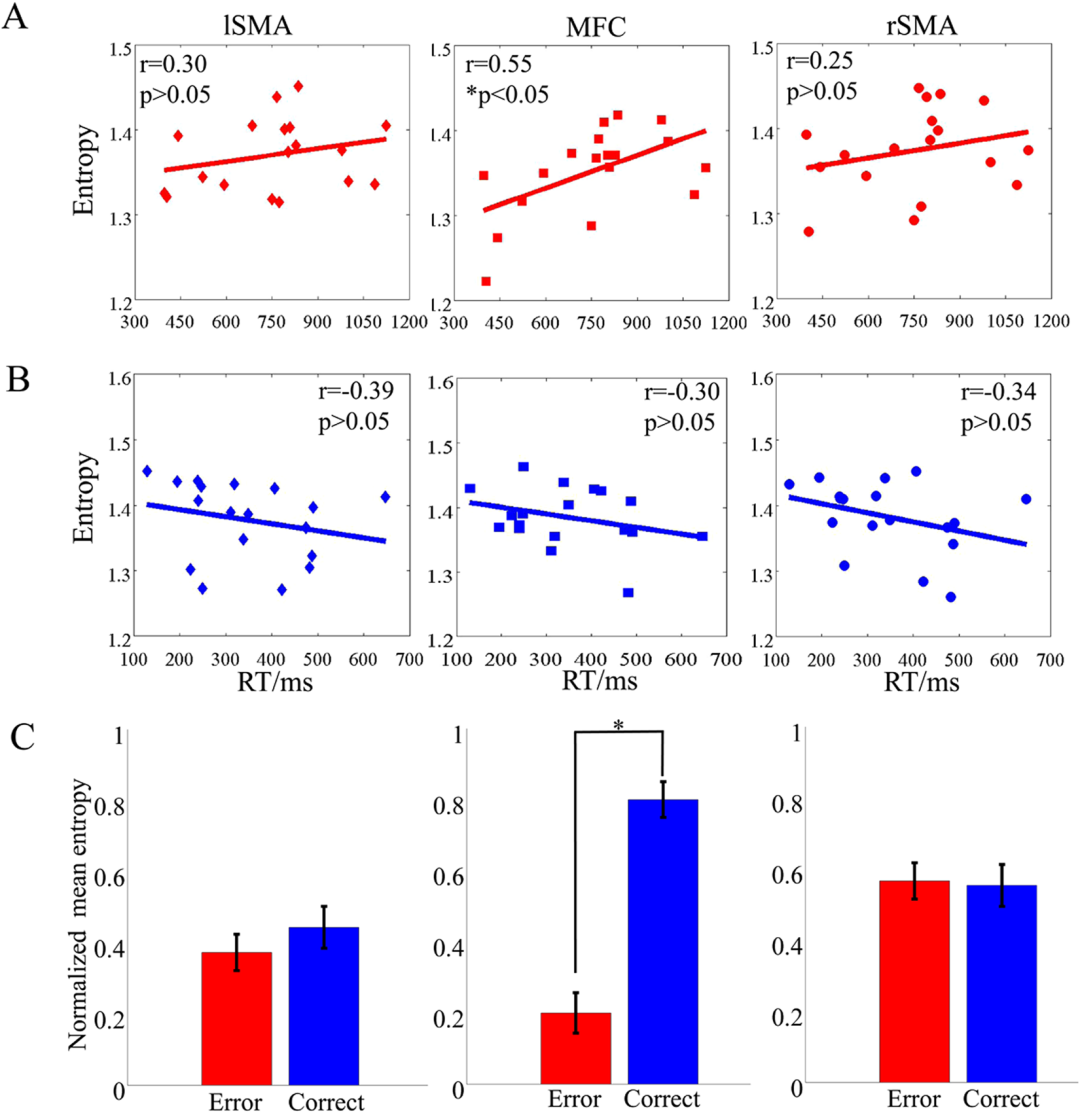
The correlations between RhEn in discriminative regions and RT. (**A**) In error trials; (**B**) In correct trials; (**C**) Normalized mean entropy (with SD) of subjects. lSMA: left supplementary motor area; MFC: medial frontal cortex; rSMA: right supplementary motor area.

RhEn of the MFC induced by error processing (i.e. error trials) was significant positively correlated with RT. RhEn of the lSMA and rSMA induced by error processing were non-significantly correlated with RT, respectively (Fig. 3A). No correlations were observed during correct response between RT and RhEn of each discriminative brain region (Fig. 3B). More detailed information was also shown in Table 3.

**Table 3:** Correlation Analysis between RT and entropy and Pair t-test between error response and correct response in discriminative regions.

Correlation	Err. (RT vs. En.)		Cor. (RT vs. En.)		Err.	Cor.	Paired t-test	
Brain region	r	p	r	p	ME.	ME.	t	p
MFC	**0**.**550**	**0**.018*	−0.305	0.219	0.200	0.800	**−3**.**141**	**0**.005*
lSMA	0.260	0.298	−0.242	0.334	0.373	0.443	−0.404	0.691
rSMA	0.252	0.313	−0.338	0.170	0.566	0.554	0.085	0.933
MFC + lSMA	**0**.**490**	**0**.039*	−0.408	0.093	0.275	0.645	**−2**.**481**	**0**.023*
MFC + rSMA	0.435	0.071	−0.411	0.090	0.322	0.718	**−2**.**647**	**0**.016*
lSMA + rSMA	0.270	0.278	−0.302	0.224	0.449	0.487	−0.261	0.797
MFC + lSMA + rSMA	0.411	0.091	−0.392	0.108	0.339	0.625	**−2**.**115**	**0**.048*

Note: Err. represents error response, Cor. represents correct response, En. is the entropy value and ME. represents normalized mean entropy of all subjects.

### Information flow between the discriminative regions

**Figure 4:**
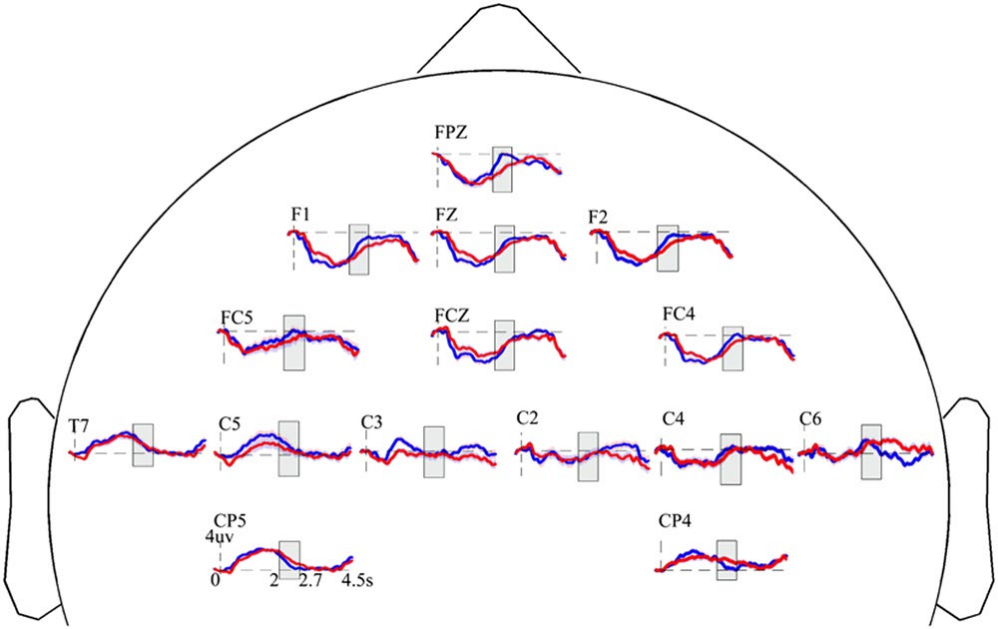
Grand average ERP waveforms at anterior electrodes. The red line represents the ERPs of error trials and blue line represents the ERPs of correct trials. Grey rectangles represent the chosen time windows, which extracted for the time period beginning auditory stimulus offset and lasting until 700 ms after auditory stimulus offset.

**Figure 5:**
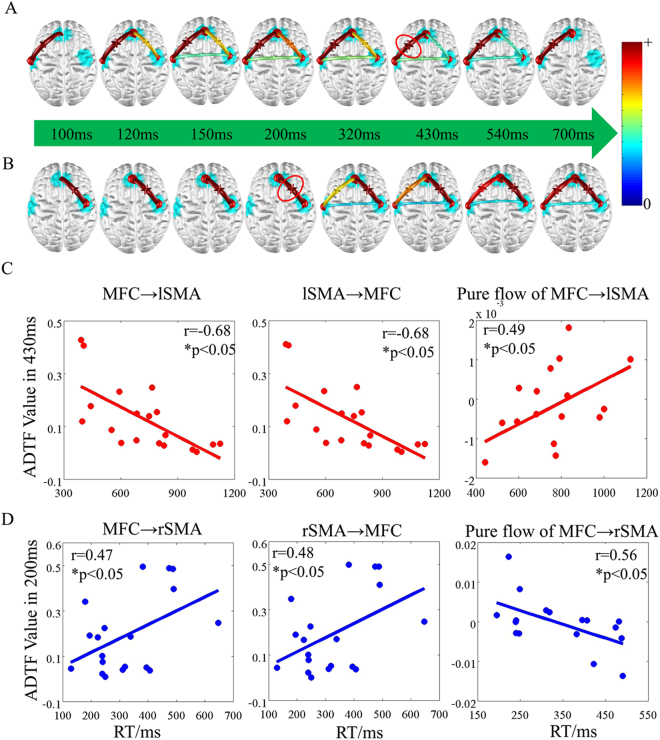
Time-varying networks and the correlations between information flow and RT. (**A**) Time-varying networks in error trials; (**B**) Time-varying networks in correct trials; (**C**) Time-varying network at 430 ms in error trials; (**D**) Time-varying network at 200 ms in correct trials.

To investigate time characteristics of two response types, i.e. correct and error, time-varying networks were conducted. Here, significant discriminative regions, i.e. the medial frontal cortex (MFC), left supplementary motor area (lSMA) and right supplementary motor area (rSMA), were utilized to serve as network nodes. For time series of networks was estimated by cortical signals within three discriminative regions, which calculated by the averaged scalp ERPs (Fig. 4). Results of the time-varying network analysis were illustrated in Fig. 5A and B.

The error time-varying networks showed the left-bias information flows between the lSMA and the MFC (i.e. lSMA → MFC and MFC → lSMA, Fig. 5A). Then, right information flow between rSMA and MFC appeared, while the correct time-varying networks showed the right-bias information flow between the rSMA and the MFC (Fig. 5B).

### Relationship between RT and information flow

Information flows between the network nodes changed with time varying. During error processing, the weaker DTF values of information flow between the medial frontal cortex and the left supplementary motor area, i.e. MFC → lSMA and lSMA → MFC, of time-varying networks at 430 ms were related to longer error RT (Fig. 5C), respectively. The pure flow between the MFC and the lSMA showed an information flow from MFC to lSMA, positively being correlated with error RTs. During correct processing, the stronger DTF values from the MFC to the right SMA, i.e. MFC → rSMA and rSMA → MFC, of time-varying networks at 200 ms were related to longer correct RT (Fig. 5D), respectively. The pure flow between the MFC and rSMA showed an information flow from MFC to rSMA, negatively being correlated with correct RTs.

## Disscussion

Based on the scalp EEG recording, the present study utilized the RhEn of cortical signal with single-trial, calculated by weighted minimum norm solution estimation (WMNE), to identify two response types (correct vs. error) differences at the cortical level during performing the auditory-tracked tasks in multi-speakers’ environment. We found that: 1) An averaged accuracy achieved 89.7% and discriminative cortex differences mainly distributed in the medial frontal cortex (MFC), the left SMA, and the right SMA; 2) The mean RhEn for error trials was significantly lower than that for correct trials in the discriminative cortices. In addition, the larger RhEn for error trials was related to longer RT. 3) Time-varying networks analysis based on discriminative regions and averaged cortical source waveforms further revealed that error-related networks represented the left-bias information flow and the correct-related networks represented the right bias information flow.

### RhEn of discriminative cortices and RT

Our classified method successfully extracted reliable differences between correct and error response with the mean classification rate of 89.7%(with SD: ±3.6%), which was superior to previous related works^5,7,30,31^ that this method was non-invasive cortical dynamical signal with single trials of short-time duration (~700 ms EEG data).

Previous study found that the medial frontal cortex played a crucial role in producing error-related potential^4,32–34^. Activation of the MFC reflected error-related processing^35^. As shown in Fig. 2A, we also found that the discriminative cortical regions between correct processing and error processing being obtained via *R*^2^ values, i.e. the relationship between rhythm entropies and class tags, and SVM mainly focused on the medial frontal cortex, revealing that the MFC was an important brain area in monitoring function between the correct and error processing in multi-speaker environment.

Entropy was a powerful tool to quantify complexity in nonlinear dynamics of neural activities. The irregularity and unpredictability of brain activity induced by attentional selection were regarded as neural complexity related to brain functions and information processing between neurons^36^. Previous studies have found that the lower entropy meant worse behavior performance^19,37^. We also found that RT for error trials significantly longer than that for correct trials (Fig. 1), indicating that increased uncertainty induced by unsuccessfully focusing attention to the cued speaker. And the mean RhEn significantly lower for error trials in MFC than that for correct trials (Fig. 3C and Table 3), suggesting that if a listener successful focused attention on a cued speaker with correct trials, the integration of segregated neuronal groups and incoming stimuli with ongoing performance induced high complexity of cortical dynamics. While if a listener did not attend a cued speaker (i.e. error trials), the decoupling and isolation of the underlying system from external factors may lead to the lower RhEn values, consistent with the previous theory confirmed by representational mathematical models^36,38^.

In addition, the larger RhEn values following with longer RT in error trials (Fig. 3A and Table 3) represented the increased irregularity and unpredictability, while there existed a trend that smaller RhEn values were related to the longer RT in correct trials (Fig. 3B and Table 3) indicated that the decreased coupling between internal system and external factors may result in the low complexity. Therefore, the lower RhEn values at the MFC in error trials during auditory processing suggested that the decoupling and segregation between the MFC and the dorsolateral prefrontal cortex, i.e. the left and the right SMA were involved in abnormal attentional control and consequently in wrong cognitive performance as well.

Previous studied found that the posterior central gyrus might be involved in the generation of processes that activated the error related potential^39^ and left dorsal lateral frontal cortex was selectively active during error trials^40^. Our findings suggested that the contribution of the left SMA and MFC to high classification rate may be related to wrong responses. And the right SMA may mainly be relevant to correct response types due to previous findings that activation was observed in the right SMA in correct trials^35,41^.The right-bias information flow may provide an evidence to support the idea during correct response (Fig. 5B).

### Information flow via time-varying networks

As described above, converging evidence revealed that left SMA was closely correlated with error related potential^39,40^. Our results of time-varying networks displayed information flow between the MFC and the left SMA in error trials (Fig. 5A). Previous findings suggested that the error-processing system consisted of a monitoring system for detecting errors and an optimized behavior compensation system^42^. When perceiving an error existing because of failure to attend the cued speaker, the left SMA firstly sent information to the MFC, and at the same time, a feedback was received from the MFC. In the process of error handing, the MFC acted as a filter to match stimuli (i.e. error or correct feedback) and reactions^43^, and then information was sent to the right SMA. In correct trials, a right-bias information flow between the MFC and the right SMA was existed (Fig. 5B). During the period from 320 ms to 540 ms, similar time-varying network connectivity patterns were observed in both correct and error trials, implying the underlying coordination of the MFC and bilateral SMA to control cognitive performance.

Moreover, during the error time-varying network at 430 ms (Fig. 5C), the smaller ADTF values of both MFC → lSMA and lSMA → MFC were related to the longer RT (Fig. 5C, left and middle panels). The pure flow showed MFC → lSMA was positively correlated with RT (Fig. 5C, right panel). During the correct time-varying network at 200 ms (Fig. 5D), the bigger ADTF values of both MFC → rSMA and rSMA → MFC were related to the longer RT (Fig. 5D, left and middle panels). The pure flow showed MFC → rSMA was negatively correlated with RT (Fig. 5D, right panel). These findings suggested that the coupling with MFC and lSMA was weaken leading to increased RT in error trials. And the coupling with MFC and rSMA was strengthened resulting in shorter RT in correct trials.

### BCI Application

In the present study, cortical activities could provide more precise spatial physiology information compared to scalp EEG^21^; Compared to traditional entropy methods such as approximate entropy, RhEn has an ability to incorporate individual’s cognitive state^27^. As shown in Table 2 and Fig. 2B, a short time duration (~700 ms) was enough to distinguish individual’s error states using the present method, which allowed the possibility of near real-time EEG processing. Using time-varying network analysis, time-varying network at 430 ms was related to error processing in wrong response and time-varying network at 200 ms was related to correct processing in right response (Fig. 5), which both were earlier than individual’s RT. These findings suggested a possible role for improving BCIs performance in the future. It was noted that for test samples, the time cost of one trial calculation was about 3 s under the MATLAB platform. Here, the most time-consuming part was the minimum norm solution estimation which taken about 0.9 s for each calculation. In the future BCI application, the efficiency of operation could be improved by using faster programming language and optimized algorithms.

## Conclusion

The present study was the first to utilize a feature extraction method, rhythm entropy coming from single cortical EEG signals with short-time duration(~700 ms) which were converted via WMNE from scalp EEG recordings during auditory processing in multi-speakers’ environment, to investigate error processing. Three brain areas, i.e. MFC, left SMA and right SMA, got high classification rate reflecting cortical discriminative source distribution between error processing and correct processing. Time-varying networks further revealed that information flows changed between these brain areas with time, i.e. time-varying network at 430 ms was related to error processing in wrong trials and at 200 ms was related to correct processing in right trials. Taken together, these findings suggested that the reduced cognitive performance on auditory error response was associated with impaired cortical information processing, as indicated by the lower complexity of the EEG.

## Material and Methods

### Participants

Twenty subjects (mean ± standard deviation (SD) age, 22 ± 3.5 years; all males; right-handed) took part in the experiment. None of them reported any history of hearing impairment or neurological problems. Informed consent was signed prior to the study, and subjects also received a monetary compensation after experiments. All experiments were approved by the ethical committee of Chongqing university of Posts and Telecommunications. All experimental methods were conducted in accordance with the ethical guidelines determined by the National Ministry of Health, Labour and Welfare and the Declaration of Helsinki (BMJ 1991; 302:1194).

### Stimuli and Design

The experiment design was similar with the previous study^4^. A sentence contained in the form [ready “call sign” go to “color” “number” point now]. For example, ready “skylark” go to “blue” “four” point now. Here, 60 unique sentences were combined by two call signs (sparrow or skylark), three colors (red, blue or green) and three numbers (two, five or seven). All sentences were read by using Chinese.

Before the experiment, subjects firstly listened to each of speakers alone and were able to report the color and number with at least 100% accuracy. In the experiment, a fixation cross (0.5° × 0.5°) at the center of the monitor were displayed throughout the entire block of trials. Each trial began with the fixation cross flashing for 50 ms. After a 700 ms delay, a cue was presented for 50 ms. The cue was defined as a call sign that tracked by listeners. After a short (100–300 ms) SOA, two different sentences spoken by a male and a female speaker were simultaneous presented about 2 s: one to the left ear, and the other to the right ear. Subjects were required to attend to one sentence, which the call sign was cued, and responded to the point where the call sign bird would go. The point (i.e. color-number combination) was fixed and shown visually on a monitor during each trial block.

### Scalp EEG recording and preprocessing

**Figure 6:**
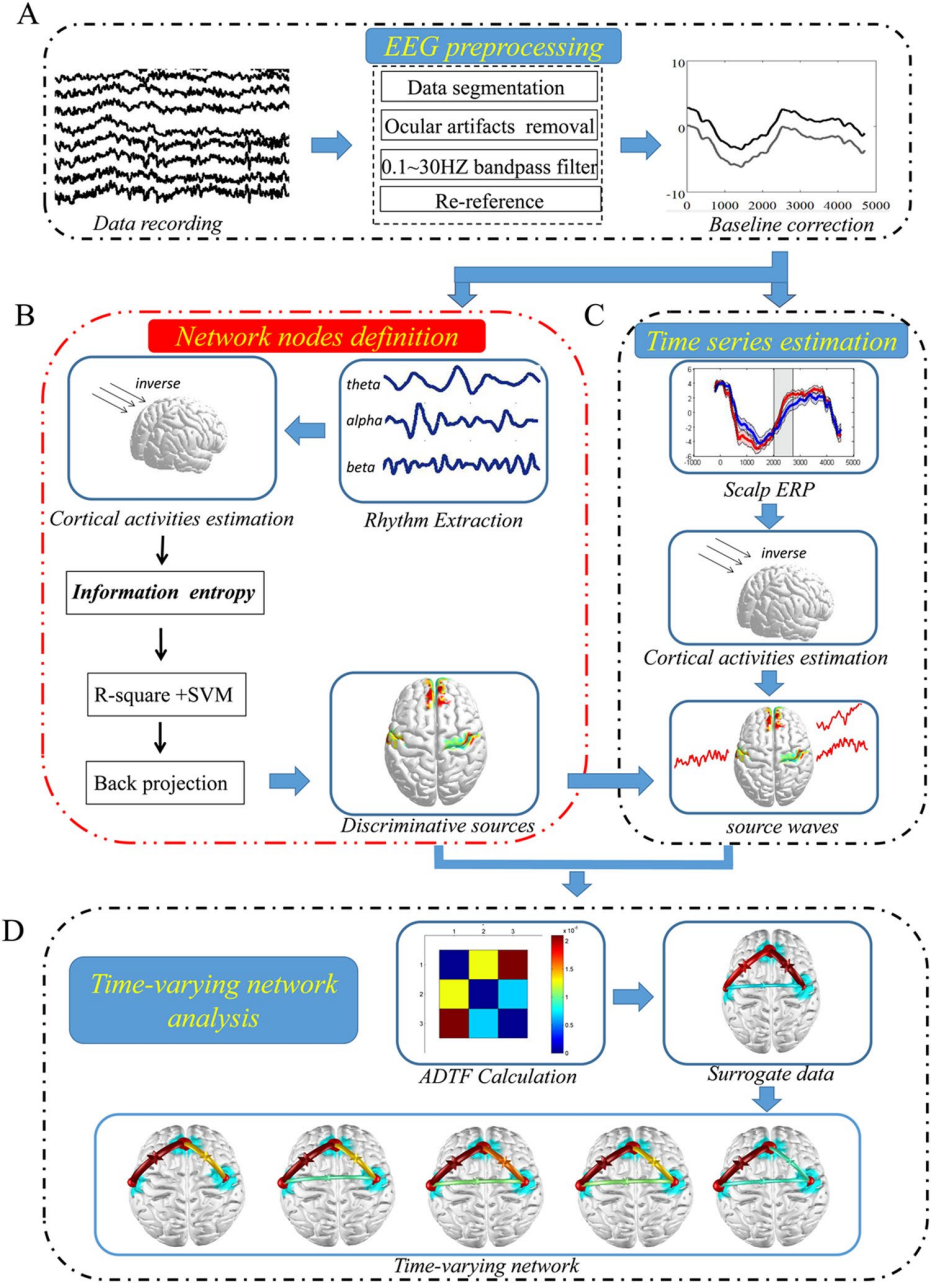
EEG analysis procedure. (**A**) EEG preprocessing; (**B**) Network nodes definition; (**C**) Time series estimation; (**D**) Time-varying network analysis.

EEG was recorded using a 64-channel NeuroScan system (Quik-Cap, band pass: 0.05–100 Hz, sampling rate: 1000 Hz, impedances <5kΩ) at the scalp. The EEG analysis procedure was shown in Fig. 6. To monitor ocular movements and eye blinks, EOG signals were simultaneously recorded from four surface electrodes, one pair placed over the higher and lower eyelid and the other pair placed 1 cm lateral to the outer corner of the left and right orbit. Cz was used as the reference during recording online. Then, the EEG recordings were divided into epochs (200 ms pre- to 4500 ms post-stimulus onset). Trials with blinks and eye movement were rejected offline and an artifact criterion of ±75 μV was used at all the other scalp sites to reject trials with excessive electromyography (EMGs) or other noise transients. EEG recordings were filtered with a band-pass of 0.1–30 Hz. The data were re-referenced by reference electrode standardization technique^44^ (REST, www.neuro.uestc.edu.cn/rest) (Fig. 6A). EEG epoching was then extracted for the time period beginning auditory stimulus offset and lasting until 700 ms after auditory stimulus offset, and performed the next analysis (Fig. 6B). Then, single-trial EEG epochs were sorted according to response types, i.e. correct-related and error-related, and were averaged from each subject to compute the ERPs (Fig. 6C).

### Network nodes definition (discriminative pattern)

Network nodes definition involved the following main steps (Fig. 6B): 1) cortical activities estimation; 2) Information Entropy; 3) R-square analysis; 4) SVM and 5) Back projection.

#### Cortical activities estimation

The conventional source localization procedure, weighted minimum norm estimation (WMNE), was used to estimate the cortical activities. For single-trial EEG epochs, three frequency-bands, i.e. theta (4–8 HZ), alpha (8–13 HZ), beta (13–30 HZ), were separately extracted by the wavelet transformation. Then cortical activities were calculated by applying linear inverse operator *W* to the three frequency-band signals:

1 $${\rm{S}}({\rm{t}})={\rm{W}}x({\rm{t}})$$

where *x*(t) represented the n-channels EEG data at time t and S(t) denoted corresponding cortical activities. *W* was obtained by:

2 $${\rm{W}}={\rm{R}}{A}^{T}{(AR{A}^{T}+{\lambda }^{2}C)}^{-1}$$

Here, C and R referred to covariance matrices of the noise and sources, respectively. A was the gain matrix, calculated via the Brainstorm toolbox (http://neuroimage.usc.edu/brainstorm/), and the regularization parameter, ${\rm{\lambda }}$, was calculated by:

3 $${\rm{\lambda }}=\frac{trace(AR{A}^{T})}{trace(C)\,\ast \,SN{R}^{2}}$$

A fixed value of 5 was used for the signal-to-noise ratio (SNR), which reflected the value in the evoked response experiments^45^.

Here, a 3-shell realistic head model was adopted for EEG source activities estimation, where the conductivities for the cortex, skull, and scalp were 1.0 Ω^− 1^ m^− 1^, 1/80 Ω^− 1^ m^− 1^, and 1.0 Ω^− 1^ m^− 1^respectively. The solution space was restricted to the cortical grey matter, the hippocampus, and other possible source activity areas, consisting of 15002 cubic mesh voxels with 10 mm inter-distance. The lead field matrix was calculated by the boundary element method (BEM)^46^.

#### Rhythm Entropy

After acquiring the cortical activities *S*(t), the power of cortical activities in each trial was then calculated by the following equation:

4 $${\rm{Power}}=\sum _{t=1}^{m}S{(t)}^{2}$$

where *m* was the number of sample points and the information entropy was measured as follow:

5 $${\rm{iEn}}=-\sum _{i=1}^{3}{P}_{i}lo{g}_{2}({P}_{i})$$

where ${P}_{i}$ (*i* = 1, 2, 3) represented the normalized power of power_i_ and was calculated via divided by the sum of three frequency-band powers, i.e. theta, alpha and beta:

6 $${P}_{i}=\frac{Powe{r}_{i}}{{\sum }_{i=1}^{3}Powe{r}_{i}}$$

#### R-square analysis

*R*^2^ analysis was a common criterion of separability in BCIs research^47^, and often used to indicate correlation between features and class tags:

7 $${R}^{2}=\frac{{(E{X}_{+1}-E{X}_{-1})}^{2}}{4{\sigma }_{X}^{2}}$$

where ${X}_{+1}$ presented feature vector of target and ${X}_{-1}$ represented feature vector of non-target. ${\sigma }_{X}$ was the standard deviation. The *R*^2^ value reflected the difference in the power of the two classes, with the larger *R*^2^ value denoting the greater difference between two classes^37^. For determining the threshold for further SVM classification, ten thresholds, i.e. from 0.1 times the maximum *R*^2^ value to the maximum *R*^2^ value and step length was set to 0.1, were selected to evaluate the performance of SVM classification. The final threshold was set to 0.6 times of the maximum *R*^2^ value among all dipoles because most of the subjects got the best classification rate under this threshold in the present study^48,49^. The dipoles with *R*^2^ value exceeding the threshold were chosen for further SVM classification.

#### Support vector machine (SVM)

In the experiment, the number of correct trials was greater than that of error trials (ACC > 80%, i.e. correct trials > 96, error trials < 24). For each subject, the number of error trials was at least 20 to ensure the training sample size. Correct trials were randomly selected to make it consistent with the amount of error trials.

SVM was developed by Vapnik based on statistics learning theory (SLT). As its excellent generalization performance, SVM has been applied in a wide variety of issues. SVM had the feature of empirical risk minimization (ERM) and global optimum solution^50^. We trained a SVM classifier with radial basis kernel function to extract highly discriminative brain regions. The goal of a SVM classifier with RBF kernel was to find a decision function ${\rm{f}}({\rm{x}})=w^{\prime} \varnothing (x)+b$ by solving the following optimization problem^51^:

8 $$\begin{array}{c}\mathop{\min }\limits_{w,\varepsilon }\frac{1}{2}||w|{|}^{2}+C\sum _{i=1}^{N}{\varepsilon }_{i}\\ {\rm{s}}.{\rm{t}}.{y}_{i}(w^{\prime} \varnothing ({x}_{i})+b)\ge 1-{\varepsilon }_{i}\end{array}$$

where *w* was the normal of the hyperplane; the function $\varnothing$ mapped the vector ${x}_{i}$ in a higher dimensional space^52^; ${\varepsilon }_{i}$ was a measure of the misclassification errors for non-separable cases; and C traded off the empirical risk and model complexity, and was set by grid search algorithm^53^. Here, C ranged from 10^−8^ to 10^8^ and the step length was set to 10^0.8^.

If the SVM classifier could reflect the relationship between features and the class labels very well, the classifier was considered that it could predict the classes of new samples with good performance. Therefore, classification accuracy (CA), sensitivity (SE), specificity (SP) and area under ROC curve (AUC) were utilized to evaluate the classification performance of SVM classifier^54^. At the same time, leave-one-out cross-validation (LOOCV) was applied to evaluate the generalization performance of SVM for a small sample size.

The percentage of the number of samples predicted correctly in the test set over the total samples, CA, was calculated as follows:

9 $${\rm{CA}}=\frac{TP+TN}{TP+TN+FP+FN}\,$$

where true positive (TP) was the number of positive samples correctly predicted and true negative (TN) was the number of negative samples correctly predicted. False positive (FP) denoted the number of positive samples incorrectly predicted and false negative (FN) denoted the number of negative samples incorrectly predicted.

SE and SP were calculated by the following formula, respectively:

10 $${\rm{SE}}=\frac{TP}{TP+FN}$$

11 $${\rm{SP}}=\frac{TN}{TN+FP}$$

SE referred to the ratio of correctly classified positive samples to the total population of positive samples, whereas SP was the ratio of correctly classified negative samples to the total population of negative samples.

#### Back projection

Both *R*^2^ analysis and SVM classifier were used to differentiate between correct-response related and error-response related brain regions (i.e. the positions of dipoles), each feature influenced the classification via its *R*^2^ value. The larger the *R*^2^ value was, the greater it affected the final discrimination. However, the correlational vector *R*^2^ was in a dimension-reduced space. To determine the discriminative brain areas, *R*^2^ values were mapped back to the high-dimensional space (i.e. dipole space). The correlational vector in the dimension-reduced subspace can be projected back to the original feature space according to the following formula:

12 $$Di=Ui\ast {R}^{2}$$

For a given dipole *i*, the correlational representation was ${D}_{i}$ and the identity matrix was *Ui* in current study. Finally, the correlations were reconverted into the MNI space to obtain the discriminative regions. A threshold was required to determine brain areas that had significantly distinct characteristics between the correct- and error-response trials. For each dipole, a statistically meaningful threshold was derived by using 0.6 times of the maximum *R*^2^ value among all dipoles, because most of the subjects got the best classification rate under this threshold in the present study.

### Time series estimation

For averaged ERPs at scalp, cortical activities (i.e. cortical ERPs) were estimated via WMNE (details described in the above section “*Cortical activities estimation*”). The time series in the discriminative brain regions localized in the above session of “network nodes definition” (Fig. 6B), were computed via averaging cortical activities of the dipoles within sources respectively (Fig. 6C).

### Time-varying network analysis

After obtaining discriminative sources (Fig. 6B) and time series in discriminative sources (Fig. 6C), time-varying network analysis was performed (Fig. 6D).

#### ADTF calculation

The multivariate adaptive autoregressive (MVAAR) model of source waves was computed by the following equation:

13 $$X(t)=\sum _{k=1}^{p}w(k,t)X(t-k)+\varepsilon (t)$$

where *X(t)* represented the cortical source wave over the entire time window, *w(k*, *t)* was the coefficients matrix of the time-varying model, which calculated by the Kalman filter algorithm, and $\varepsilon (t)$ represented the multivariate independent white noise. The symbol *p* denoted the MVAAR model order selected by Schwarz Bayesian Criterion^28,55^.

As mentioned above, the discriminative brain areas as the cortical sources (Fig. 6B) were applied in the time-varying network analysis. After obtaining the MVAAR model coefficient (*w(k*, *t))*, *H(f*, *t)* was obtained from *w(f*, *t)*, which  was then transformed by Eq. (13) into the frequency domain. The *H*_*ij*_ element of *H(f*, *t)* described the directional information flow between the *j*th and the *i*th element at each time point *t* as:

14 $$w(f,t)\ast X(f,t)=\varepsilon (f,t)$$

15 $$X(f,t)={w}^{-1}(f,t)\ast \varepsilon (f,t)=H(f,t)\ast \varepsilon (f,t)$$

where $w(f,t)=\sum _{k=0}^{p}{w}_{k}(t){e}^{-j2\pi f\Delta tk}$, *w*_k_ was the matrix of the time-varying model coefficients. $X(t)$ and $\varepsilon (t)$ were transformed into the frequency domain as *X(f,t)* and $\,\varepsilon (f,t)$, respectively.

Defining the directed causal interrelation from the *j*th to the *i*th element, the normalized ADTF was described between (0, 1) as follows,

16 $${\iota }_{ij}^{2}(f,t)=\frac{|{H}_{ij}(f,t){|}^{2}}{{\sum }_{k}^{n}|{H}_{ik}(f,t){|}^{2}}$$

To obtain the total information flow from a single node, the integrated ADTF was calculated as the ratio of summation of ADTF values divided by the interested frequency bands [*f1*, *f2*]:

17 $${\vartheta }_{ij}^{2}(t)=\frac{{\sum }_{f1}^{f2}{\iota }_{ij}^{2}(k,t)}{f2-f1}$$

We chose average ADTF values over 4–30 Hz to acquire the final directional information flow according to the range of three frequency bands.

#### Surrogate data testing

The distribution of ADTF estimator under the null hypothesis of no causal interactions was not well determined, since the ADTF function had a highly nonlinear correlation with the time series where it derived. In view of this, the phases of the Fourier coefficients were independently and randomly iterated to produce a new surrogate data, which was a nonparametric statistical test^28^. The spectral structure of the time series was retained in the process of iterating the phases of the Fourier coefficients. The shuffling procedure was repeated 200 times for each model-derived time series of each subject to establish an empirical distribution of the ADTF value under the null hypothesis of no connectivity.

### Correlation analysis

We performed Pearson correlation analysis to investigate the following relationships: 1) RT and entropy values of discriminative regions; and 2) RT and ADTF values of information flow between discriminative regions. All thresholds were set at p < 0.05. Here, ADTF value of information flow was adjusted by dividing the weighted degree of network.
